# Endocrine Signaling and Tissue Remodeling in Gynecomastia: Implications for Treatment Response

**DOI:** 10.3390/ijms27219443

**Published:** 2026-10-23

**Authors:** Jian Deng, Shanshan Tong, Siqi Liu, Haisheng Zhou, Min Ren

**Affiliations:** 1 Department of Breast Surgery, The First Affiliated Hospital of Anhui Medical University, Hefei 230022, China; 2546010177@stu.ahmu.edu.cn; 2 Faculty of Chinese Medicine, Macau University of Science and Technology, Avenida Wai Long, Taipa, Macao 999078, China; 3250007803@must.edu.mo; 3 The First Clinical Medical College, Anhui Medical University, Hefei 230032, China; 2413010329@stu.ahmu.edu.cn; 4 Department of Biochemistry and Molecular Biology, Anhui Medical University, Hefei 230032, China

**Keywords:** gynecomastia, treatment response, estrogen receptor, tissue remodeling, ultrasonography, patient-derived organoids

## Abstract

To critically synthesize evidence on treatment response in gynecomastia using a framework that distinguishes biological response, structural response, and patient-important benefit. We searched PubMed, PubMed Central, and targeted journals through 11 September 2026. Priority was given to gynecomastia-specific human studies; evidence from other breast systems, animal models, and general biomarker methodology was treated as indirect. The randomized anastrozole trial showed an endocrine effect but did not demonstrate superiority over placebo on the prespecified structural endpoint. Receptor expression in surgical specimens provides biological plausibility but does not establish receptor functionality, endocrine dependence, or predictive value. Stromal composition may influence lesion regression, but this remains a hypothesis requiring direct testing. Ultrasonography and elastography may support pretreatment characterization, but current studies do not validate imaging features as surrogates for fibrosis or endocrine responsiveness. Gynecomastia-derived organoids offer a disease-relevant model for functional studies, although donor numbers are small and endocrine responsiveness, cellular representativeness, and reproducibility across patients require further evaluation remain uncertain. Biological response, structural response, and patient-important benefit need not occur together or in a fixed sequence. Further research should link tissue and model findings to pretreatment markers, compare treatment benefits, and establish clinical utility before marker-guided care.

## 1. Introduction

Gynecomastia is a benign proliferation of glandular and stromal tissue in the male breast, rather than merely increased breast prominence. Clinical assessment distinguishes glandular enlargement from predominantly adipose enlargement and other breast abnormalities, while considering endocrine, systemic, and medication-related causes. Symptom duration, discomfort, severity, and patient preferences guide the choice between observation, treatment of an underlying disorder, pharmacological therapy, and surgery [1,2]. Earlier examination-based studies identified gynecomastia in otherwise healthy adolescents and adults, whereas a recent study of approximately 530,000 boys captured clinically recorded cases rather than all palpable glandular enlargement [3,4,5]. These differences in case ascertainment matter when interpreting findings on natural history and treatment response.

Patients with similar degrees of breast enlargement may differ in the factors sustaining the condition, tissue composition, and treatment goals. These differences matter when interpreting findings from translational studies. Surgical tissue collections and referral clinic cohorts may not represent the broader population, particularly patients whose gynecomastia regresses spontaneously or who receive no intervention. Molecular findings from these selected cohorts should be considered in the context of the patients from whom they were obtained, not assumed to apply to all patients with gynecomastia.

Clinical benefit extends beyond a reduction in breast size. Case–control and longitudinal studies have documented the psychological and functional burden associated with gynecomastia [6,7,8]. Symptom relief, glandular regression, improved chest contour, and patient satisfaction are distinct outcomes and may not change in parallel. A treatment may alter endocrine signaling without reversing established structural changes, while an improvement in chest contour may still fall short of a patient’s expectations. A marker associated with one outcome may provide little information about another.

Recent tissue profiling studies and the development of gynecomastia-derived organoids offer opportunities to investigate the biology underlying these clinical differences [9,10]. The clinical relevance of these findings cannot be inferred solely from experience with receptor-directed treatment in breast cancer. A prognostic biomarker provides information about the likely course of a condition, whereas a predictive biomarker identifies differences in benefit between treatment options. Demonstrating predictive value requires an appropriate treatment comparison; establishing clinical utility further requires evidence that using the marker improves treatment decisions [11,12]. This review uses that distinction to evaluate the emerging evidence and its relevance to treatment selection in gynecomastia.

Existing gynecomastia guidelines primarily address etiological evaluation and management, whereas previous reviews have focused mainly on pathophysiology, natural history, or pharmacological options. This review addresses a related but distinct question: how should evidence be interpreted when biological activity, structural change, and patient-important benefit do not occur in parallel? We place tissue signaling, stromal remodeling, imaging, and patient-derived models within a translational framework for evaluating pretreatment markers and treatment-effect modification. The framework is hypothesis-generating rather than a validated clinical classification or treatment algorithm. Surgery is considered as a comparator and as a source of tissue for translational studies; technical comparisons of surgical procedures are outside the scope of this review.

## 2. Conceptual Framework and Review Scope

### 2.1. Three Complementary Outcome Domains

Treatment response can be assessed across three related but distinct domains. Biological response refers to changes in receptor-dependent transcription, local steroid conversion, or epithelial proliferation. Structural response refers to measurable changes in glandular tissue, stroma, or chest contour. Patient-important benefit refers to meaningful improvement in pain, appearance-related distress, satisfaction with appearance and chest contour, psychological well-being, or daily functioning.

Receptor expression, stromal composition, and imaging features are baseline measurements that may help define biological context; they are not themselves treatment responses. A biological response requires a treatment-induced change in a relevant process, whereas structural response and patient-important benefit require separate outcome assessments.

**Figure 1:**
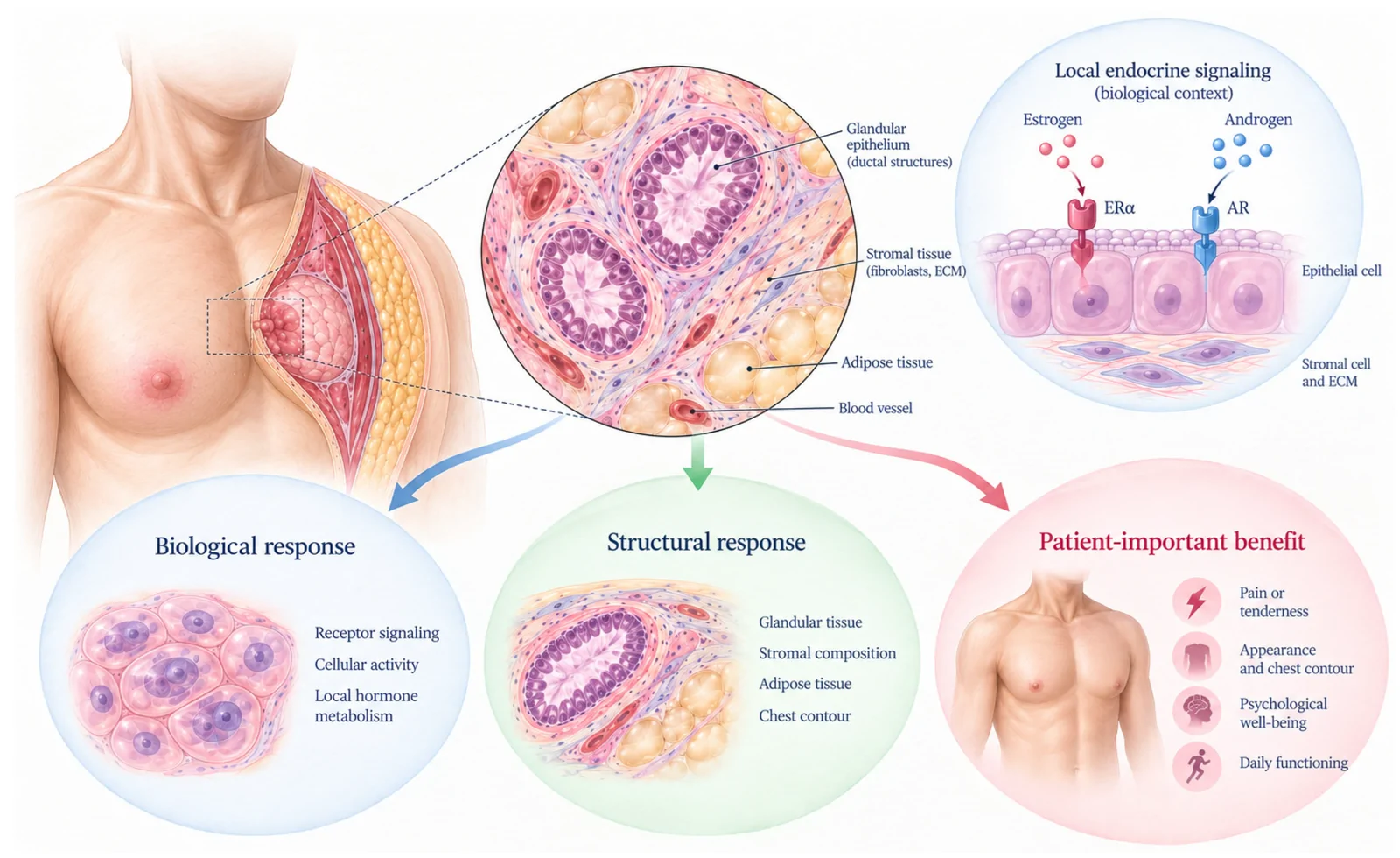
Treatment response framework in gynecomastia. Treatment response can be evaluated in three related but distinct domains. Biological response refers to treatment-induced changes in receptor-dependent transcription, cellular activity, or local hormone metabolism. Structural response encompasses measurable changes in glandular tissue, stromal composition, adipose tissue, or chest contour. Patient-important benefit includes pain relief, satisfaction with appearance and chest contour, psychological well-being, and daily functioning. Improvements in one domain do not necessarily translate into improvements in the others. The image is a schematic illustration; no panel represents original patient or experimental data. ERα, estrogen receptor alpha; AR, androgen receptor; ECM, extracellular matrix.

These domains are complementary and need not follow a fixed sequence. Symptoms may improve without documented glandular regression, and a reduction in tissue volume does not necessarily mean the patient has benefited. Structural reversibility describes a proposed tissue property, whereas structural response is an observed treatment outcome. The framework is intended to guide interpretation of the evidence, not to serve as a validated staging system or suggest that all patients require tissue testing (Figure 1).

Akgül and colleagues reported post-treatment histological findings in three adolescents who remained dissatisfied after tamoxifen treatment and subsequently underwent surgery. The resected specimens contained adipose tissue without intraductal epithelial proliferation [13]. Without paired pretreatment and post-treatment evidence or an appropriate comparator, these selected cases cannot establish that tamoxifen eliminated pre-existing glandular disease. They do, however, raise the possibility that the tissue component affected by a drug may differ from the component contributing to persistent dissatisfaction. A simple designation of treatment success or failure may be insufficient to guide subsequent management.

The timing and context of measurements also matter. Fibrosis observed in a specimen obtained after unsuccessful medical treatment does not establish that it could have predicted treatment failure. Such a claim would require evaluation based on pretreatment measurements. Similarly, a drug response in culture does not by itself establish that a patient will benefit from the same treatment. Interpretation of a translational finding depends on when the measurement was made, the biological compartment examined, the comparator used, and the outcome assessed.

### 2.2. Review Scope and Evidence Sources

This critical narrative review was based on focused searches of PubMed and PubMed Central, supplemented by targeted searches of relevant journal publications and reference tracing. During revision, supplementary source verification was performed on 9 October 2026 using web searches restricted to https://pubmed.ncbi.nlm.nih.gov and https://pmc.ncbi.nlm.nih.gov. The web-search queries were “gynecomastia tamoxifen”, “gynecomastia organoids”, “gynecomastia histology receptor aromatase fibrosis”, and “gynecomastia ultrasonography elastography”. Publisher pages were also consulted where accessible. No explicit language filter was applied to these supplementary web searches. Literature published through 11 September 2026 was considered. Priority was given to primary human studies specific to gynecomastia. Evidence drawn from female breast tissue, breast cancer, animal models, and general biomarker methodology was used as indirect mechanistic or methodological context and is identified as such in the text.

For Table 1 and Table 2, studies were selected as representative when they addressed a distinct etiologic, tissue, imaging, therapeutic, or model-based question and reported enough information to interpret the population, comparison, and outcome. Comparative human studies were prioritized for treatment claims, whereas uncontrolled studies were used to describe response signals rather than comparative effects. No formal language restriction or duplicate screening was prespecified; in practice, the included literature was predominantly English-language. When only an indexed abstract was available, conclusions were limited to the information reported there. This was not a registered systematic review; exhaustive database searching, independent duplicate screening, formal risk-of-bias grading, and quantitative pooling were not undertaken. Table 1 and Table 2 summarize representative evidence and the questions that the respective study designs can address.

**Figure 2:**
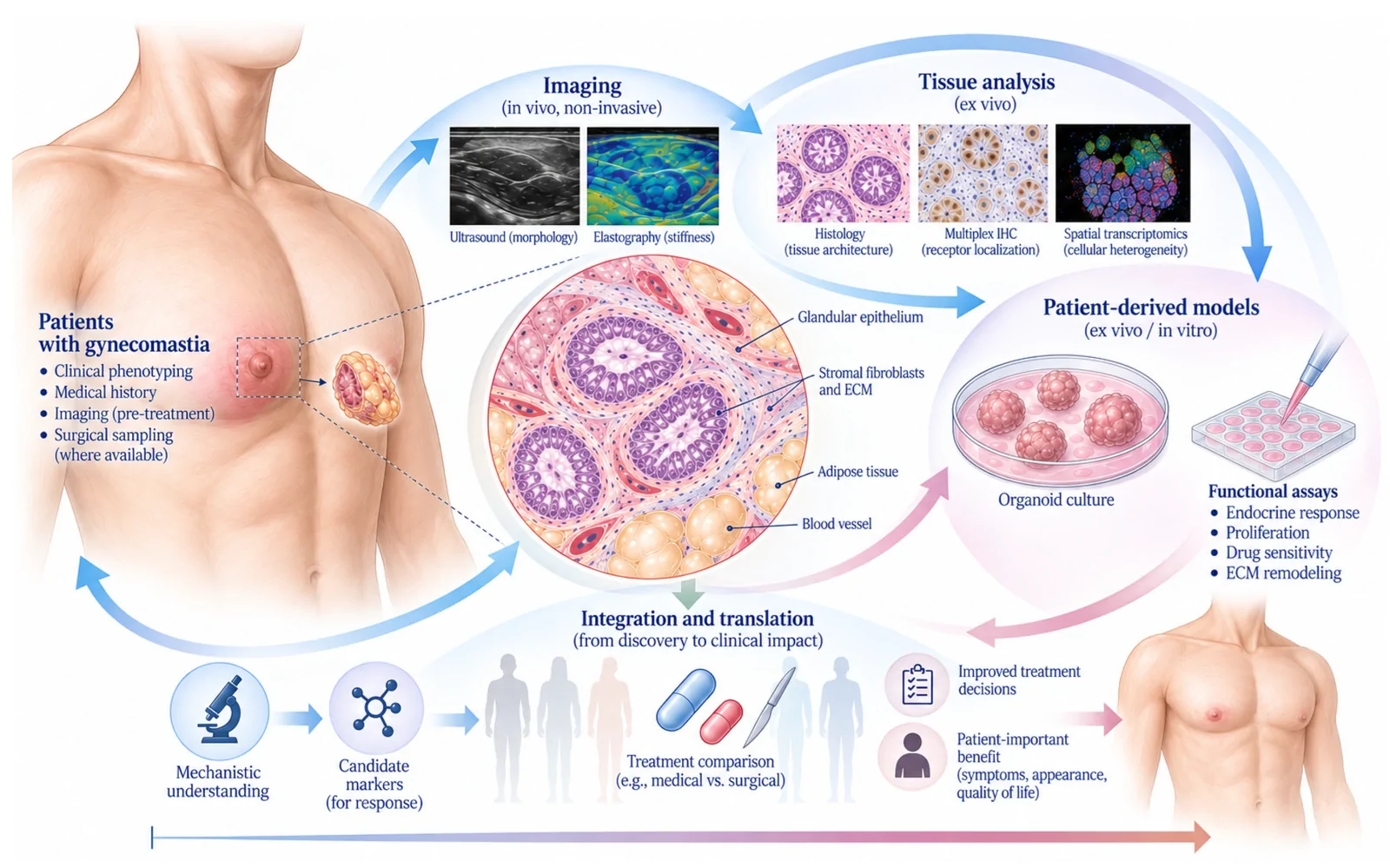
Proposed translational workflow from patients to mechanisms in gynecomastia. Clinical phenotyping and imaging may provide candidate pretreatment assessments, whereas clinically indicated surgery can provide tissue for histology, immunohistochemistry, and spatial transcriptomics. Patient-derived organoids may support proposed functional assays of endocrine response, proliferation, drug sensitivity, and extracellular-matrix remodeling, but these applications require validation. Findings are intended to generate hypotheses and inform future treatment comparisons rather than to guide current treatment selection. Blue and pink arrows indicate the respective research workflows, while the bottom gradient arrow represents clinical translation. The image is a schematic illustration; no panel represents original patient or experimental data. IHC, immunohistochemistry; ECM, extracellular matrix.

**Figure 3:**
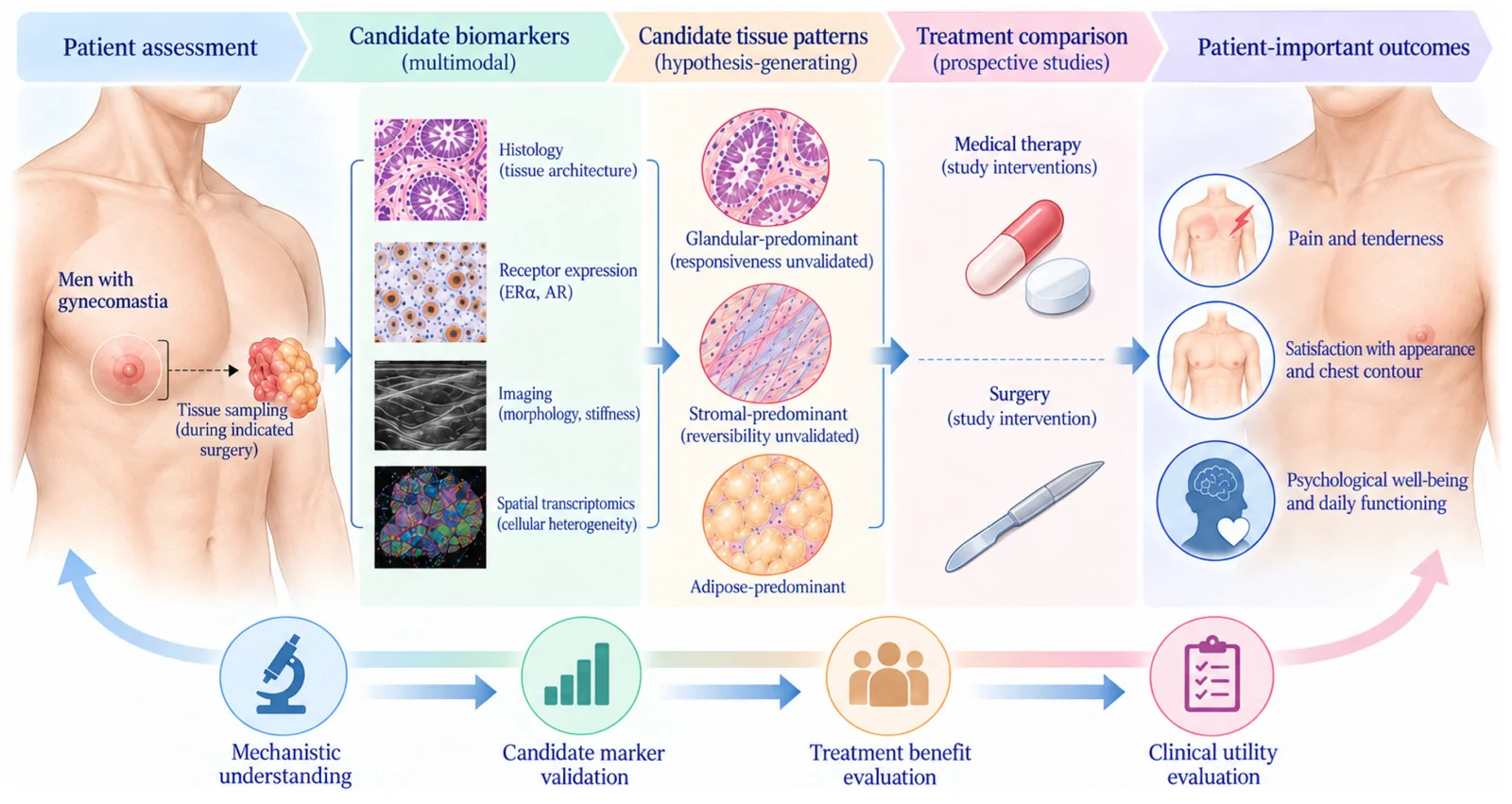
Proposed translational pathway from biomarker discovery to patient-important benefit. Histological, receptor, imaging, and transcriptomic findings can generate candidate markers and hypotheses regarding glandular-, stromal-, or adipose-predominant tissue patterns. Comparative studies are needed to determine whether these markers identify differential benefit from medical therapy versus surgery. The categories shown are conceptual and do not represent validated biological subtypes or a treatment algorithm. Tissue sampling is limited to clinically indicated surgery or discovery cohorts and is not implied for routine care. The image is a schematic illustration; no panel represents original patient or experimental data. ERα, estrogen receptor alpha; AR, androgen receptor; SERMs, selective estrogen receptor modulators.

Generative AI was used to assist in generating the schematic illustrations for Figure 1, Figure 2 and Figure 3. The authors reviewed and edited all AI-assisted outputs and take full responsibility for the final content.

To make evidence boundaries explicit, studies were categorized as primary human comparative evidence (randomized or nonrandomized), prospective or retrospective observational evidence, cross-sectional or post-treatment tissue evidence, patient-derived model evidence, or indirect evidence from other breast systems, animal models, or general methodology. These categories indicate the type of question addressed and do not represent a formal certainty-of-evidence rating.

## 3. Clinical Context and Local Endocrine Signaling

Clinical context defines the population in which a treatment is evaluated, while tissue studies identify mechanisms that may operate within that population. Causal evidence from specific endocrine disorders, descriptive receptor measurements, and tests of functional dependence provide different kinds of information. Their implications for treatment selection should be considered separately.

### 3.1. Developmental Trajectories and Persistent Stimulation

Identifying the underlying cause helps define the biological context for treatment. A study of 786 adult men and a separate multicenter clinical series document the diversity of endocrine findings and causes among referred patients [26,27]. Adult-onset disease should be distinguished from pubertal gynecomastia that persists into adulthood. Prepubertal presentation also warrants a different diagnostic emphasis, as studies of its etiology and subsequent course illustrate [28]. Combining these populations without prespecified stratification may obscure treatment effects or produce apparent biomarker associations driven by age and referral patterns.

Pubertal gynecomastia occurs during a period of changing hormone concentrations and growth. Studies of physical maturation and hormone concentrations, the temporal association with peak height velocity, and longitudinal measurements of growth, sex steroids, and insulin-like growth factor 1 (IGF-1) place the condition within this developmental context [29,30,31]. Associations with anthropometric measures in a cross-sectional study of 6200 boys provide complementary population-level evidence [32]. These findings support considering pubertal stage and growth trajectory alongside chronological age when studying treatment response. They do not, however, establish a single circulating hormone threshold that distinguishes reversible from persistent disease.

The timing of medication and other relevant exposures also matters. Epidemiological studies of 5-alpha-reductase inhibitors and prospective observations of androgen abuse have identified exposures that may alter the balance between breast stimulation and inhibition [33,34]. Experimental work with efavirenz demonstrated estrogen receptor modulation in breast cancer cells, providing a possible mechanism but not direct evidence of the same process in a patient’s benign lesion [35]. Exposure onset, continuation, withdrawal, and recurrence should be documented separately. Regression after withdrawal and improvement during continued exposure answer different questions, even when the same treatment is used for gynecomastia.

Baseline assessment should record the source and persistence of stimulation, the measurable state of the breast tissue, and the burden experienced by the patient. These should initially remain separate variables rather than be combined into an unvalidated severity score. Ongoing stimulation may coexist with modest enlargement, whereas a substantial contour abnormality may persist after the stimulus has resolved. Keeping these features distinct helps explain why hormonal treatment, structural intervention, or observation may address different aspects of the same clinical presentation.

Age and developmental status should also be treated as potential effect modifiers rather than background variables. Pubertal, persistent pubertal, adult idiopathic, medication-associated, and endocrine-disorder-associated gynecomastia differ in hormonal context, expected natural history, tissue state, and treatment goals. Evidence from one population should therefore not be generalized to another without prespecified stratification or an explicit rationale.

### 3.2. Human Genetic Evidence and Treatment Implications

Aromatase excess syndrome offers particularly informative human evidence linking altered steroid conversion to gynecomastia. Gain-of-function mechanisms involving CYP19A1, including structural rearrangements, together with biochemical observations in affected families, link a defined molecular abnormality to estrogen excess and breast development [36,37,38]. Long-term observations of aromatase inhibition in this syndrome support a mechanism-specific approach to treatment, although the outcomes assessed include growth and endocrine development as well as breast regression [39]. This experience does not establish the effectiveness of the same treatment in common idiopathic pubertal gynecomastia.

Partial androgen insensitivity provides a complementary example. Reports of tamoxifen treatment in affected siblings and management data from an international registry describe variable breast outcomes and heterogeneous treatment experience in patients with impaired androgen action [40,41]. Identifying the causal defect helps explain susceptibility, but uncertainty remains about how much established breast tissue can regress. A specific etiology improves biological interpretation without, by itself, predicting treatment response.

Candidate-gene studies address a different question. Studies of CYP19 polymorphisms, estrogen receptor beta and leptin receptor variants, and GPR30 variants have explored disease susceptibility, whereas an investigation of androgen receptor CAG-repeat length found no clear difference between cases and controls [42,43,44,45]. These findings do not establish a basis for pharmacogenetic treatment selection. A susceptibility variant may exert its effects during a transient developmental period and offer little information about the response of an established lesion. Genetic analyses should specify whether the outcome of interest is disease onset, persistence, spontaneous regression, or differential treatment benefit.

### 3.3. Circulating Hormones and Local Endocrine Signaling

The balance between estrogenic stimulation and androgenic restraint remains a useful concept, although it is not fully captured by clinical hormone measurements. Studies of eugonadal men with idiopathic gynecomastia have reported biochemical differences in the absence of overt gonadal failure [46]. Earlier experiments found increased aromatase activity in pubic skin fibroblasts from affected patients, suggesting altered peripheral steroid conversion in at least some individuals [47]. These measurements do not directly quantify steroid production within the breast. The findings provide a rationale for investigating local signaling, but do not establish a role for routine tissue assays.

Assay limitations add uncertainty to the interpretation of circulating hormone concentrations. An Endocrine Society position statement describes limitations of estradiol measurements, particularly at low concentrations [48]. The interpretation of an apparently normal result depends on assay performance, reference intervals, sampling conditions, and physiological context. An estradiol-to-testosterone ratio also combines uncertainty from two measurements and is sensitive to the units used and the choice of total or free hormone concentrations. Future studies should prioritize reproducible assays and clearly defined analytes rather than attempt to derive universal thresholds from heterogeneous retrospective datasets.

Direct studies of breast tissue offer a more anatomically relevant assessment. Prasad and colleagues obtained evaluable immunohistochemistry from 108 specimens representing 86 patients. Estrogen receptor alpha (ERα) positivity was reported in approximately 99% of evaluable specimens, whereas androgen receptor positivity was less common [9]. Singh and colleagues studied 29 men undergoing surgery for bilateral moderate-to-severe idiopathic gynecomastia and reported frequent expression of estrogen receptor (ER), aromatase, androgen receptor, and progesterone receptor, together with IGF-related findings [14]. These studies identify plausible components of local signaling. They do not establish that circulating hormone concentrations are irrelevant or that all lesions depend on the same pathway, nor do they show that a nearly universal positive stain can distinguish responders from nonresponders.

### 3.4. Receptor Expression and Cellular Localization

The receptor literature spans ligand-binding assays, immunohistochemistry, and transcript measurements [49,50,51,52,53]. Differences in sampling, fixation, antibodies, scoring methods, and positivity thresholds may account for some of the variation in reported receptor frequencies. Pooling these estimates without harmonization would treat distinct measurements as though they represented the same biological quantity. For translational studies, receptor detection is only a starting point: the cells expressing the receptor, the level of expression, and its functional consequences also need to be defined.

The epithelial architecture of gynecomastia illustrates why localization matters. Histological studies have described a three-layered ductal pattern, while broader profiling has identified mixed basal and luminal cytokeratin expression [9,54]. Bulk transcript measurements or overall staining intensity may reflect differences in cell proportions rather than changes within hormone-sensing cells. Similarly, studies of prolactin receptor expression establish the presence of a signaling component, but do not justify receptor-directed treatment in otherwise unselected patients [55].

Receptor presence, receptor functionality, endocrine dependence, and predictive biomarker value are distinct propositions. Presence refers to detection of receptor protein or transcript; functionality requires a response to ligand or a relevant perturbation; endocrine dependence requires that receptor-directed intervention alters that response; and predictive value requires a treatment comparison showing differential benefit. A positive stain therefore provides biological context but does not, by itself, identify patients who will benefit from endocrine therapy.

Assay specificity also requires careful evaluation. An extensive antibody-validation study raised concerns about estrogen receptor beta (ERβ) detection across research settings [56]. These concerns do not establish that ERβ is absent in gynecomastia or invalidate all earlier findings. They do, however, emphasize the need for appropriate controls and confirmation by independent methods. Concordance among protein localization, transcript detection, and receptor-linked functional responses would be more persuasive than repeating an assay with another unvalidated antibody.

Studies of normal female breast tissue offer mechanistic hypotheses rather than direct evidence for gynecomastia. Steroid receptor expression and proliferation do not necessarily occur in the same cells, and single-cell analyses have identified epithelial populations with distinct signaling characteristics [57,58]. These findings suggest that hormone-sensing cells may influence neighboring cells through paracrine signaling. Such a mechanism could explain why receptor abundance and proliferation do not always change together, but it requires direct testing in male gynecomastia. Pathways described in female mammary tissue or malignant cells should be treated as hypotheses when applied to this condition.

### 3.5. Toward a Functional Endocrine Phenotype

A functional endocrine phenotype could combine receptor localization, local steroid-conversion capacity, downstream signaling activity, and the identity of the responding cells. High receptor expression accompanied by low proliferation might indicate a hormone-responsive but relatively stable lesion, rather than complete endocrine independence or imminent regression. Conversely, low measured receptor expression might result from sampling a fibrous region or from loss of a relevant cell population during culture. Paired tissue and model studies could help distinguish these possibilities.

Functional testing requires a defined stimulus, a prespecified response, and a mechanistically relevant perturbation. Changes in proliferation, transcription, or ductal architecture should be assessed alongside cell viability and lineage composition. A reduction caused by nonspecific toxicity does not demonstrate selective endocrine dependence. A validation strategy would establish reproducible ligand responsiveness, show that receptor-directed intervention interrupts that response, and confirm the finding with an independent molecular readout. This is a proposed research strategy, not an established clinical assay. Its purpose is to move beyond descriptive expression and test mechanisms relevant to treatment response [11,12].

Growth-related signaling also warrants closer examination. The longitudinal pubertal study associated physiological gynecomastia with IGF-1 and growth, whereas the surgical tissue investigation detected IGF-1 immunoexpression in a selected adult population [14,31]. One describes a developmental trajectory; the other identifies a tissue-associated signal. Together, these observations support further investigation, but do not establish that local IGF-1 production causes persistence or that its inhibition would improve clinical outcomes. Circulating exposure, tissue uptake, cellular source, and functional dependence remain separate questions.

The same overall tissue signal may have several explanations. A stronger signal could reflect a greater number of hormone-sensing cells, increased signaling within those cells, or a higher proportion of responsive neighboring cells. A low proliferative index may also coexist with an expanded but relatively stable epithelial compartment. Cell-resolved measurements would help distinguish these possibilities more effectively than bulk expression profiles. The next step is to specify which of these patterns predicts a response to a defined intervention and test that prediction, rather than infer a therapeutic target from cross-sectional coexpression [9,57,58].

## 4. Tissue Remodeling and Treatment Response

Whether medication reduces an established lesion may depend on stromal composition and remodeling capacity as well as receptor expression and pathway activity. The predictive value of these tissue features remains to be established. This section considers histological findings alongside therapeutic evidence, with symptom relief, structural change, prevention, and treatment-related harms assessed separately.

### 4.1. Histological State, Age, and Disease Duration

The distinction between florid epithelial–stromal proliferation and a more fibrous appearance is useful for describing tissue morphology, but does not establish a reliable timeline for an individual lesion. Fricke and colleagues evaluated 305 surgical patients, representing 555 breasts, and found fibrous appearances more frequently in adolescents and young adults than in older patients [15]. These findings challenge the assumption that younger age indicates active disease and older age indicates fibrosis. Because the study was cross-sectional and limited to surgical cases, it could not establish the natural progression of untreated gynecomastia.

Chronological age, reported duration, and tissue state should be recorded separately. Patients may date onset from when they first noticed breast prominence rather than from the beginning of biological change, and persistent enlargement may include periods of renewed stimulation. Although a duration threshold may be clinically useful, it is not a direct measure of collagen organization and cannot establish that a particular patient will not benefit from endocrine treatment. Structural reversibility needs to be assessed through a defined outcome over time, rather than inferred retrospectively after surgery.

### 4.2. Stromal Composition and Heterogeneity

Studies of gynecomastia stroma suggest that extracellular matrix composition varies across morphological states. In a study of 36 specimens, type VI collagen was prominent in florid lesions but reduced or absent in densely fibrotic regions [16]. Another investigation described predominantly CD34-positive, α-smooth-muscle-actin-negative stroma, in contrast to patterns observed in male breast carcinoma [59]. These findings indicate variation in both matrix composition and fibroblastic phenotype. The activated myofibroblast model of malignant stroma should not be assumed to apply to benign breast enlargement.

Experimental studies in mammary systems have shown that tissue tension and matrix crosslinking can alter cellular behavior and integrin-associated signaling [60,61]. These findings suggest that existing tissue architecture could influence whether an endocrine response leads to a change in breast volume or shape, although direct evidence in gynecomastia remains limited. In the 29-patient tissue study discussed above, no positive transforming growth factor beta (TGF-β) immunoexpression was detected under the assay conditions used [14]. This observation does not exclude activity of the pathway, but the available findings do not establish TGF-β-driven fibrosis as a mechanism in gynecomastia.

Across these mechanisms, direct evidence in gynecomastia is currently limited to descriptive observations of collagen distribution, stromal marker patterns, and immunoreactivity measured under specific assay conditions. Evidence that matrix tension or crosslinking alters epithelial behavior comes mainly from other mammary or cancer models and is therefore indirect. The proposed link to treatment response is that stromal organization may constrain the structural consequence of endocrine suppression. The key gap is paired pretreatment assessment of tissue architecture, treatment exposure, and patient-important outcomes.

### 4.3. Endocrine Responsiveness and Structural Reversibility

For this review, structural reversibility denotes the hypothesized, context-dependent capacity of established tissue to regress in response to altered endocrine stimulation. The term concerns biological tissue regression rather than surgical removal. Structural response, by contrast, is an observed change in a prespecified structural outcome over a defined interval. Age, symptom duration, receptor expression, and tissue morphology do not, by themselves, establish structural reversibility or treatment-predictive value. Symptom relief or changes in endocrine signaling alone do not demonstrate structural regression.

In the proposed two-component model, endocrine responsiveness refers to the capacity of tissue to alter biological processes regulated by endocrine signaling in response to a defined endocrine perturbation, whereas biological response denotes the treatment-induced change actually observed in those processes. Tissue composition and remodeling capacity are hypothesized to influence whether a biological response is accompanied by structural regression. Similar baseline receptor staining could therefore coexist with different structural responses. This remains a working hypothesis rather than an established molecular classification. The post-tamoxifen histological cases discussed above highlight the need to assess tissue findings, structural outcomes, and patient satisfaction separately; they do not validate the proposed model [13].

One approach to testing this model would be spatially matched assessment of the epithelium, periductal stroma, surrounding matrix, and adipose tissue, using specimens from clinically indicated surgery or appropriately designed research sampling. Candidate measures include epithelial area, proliferation, collagen distribution and organization, and regional tissue mechanics. Serial imaging could assess structural change, while changes in biological activity and patient-important outcomes would require separate measurements. Glandular shrinkage should be distinguished from changes in surrounding fat, skin redundancy, and overall chest contour. These are proposed research approaches, and findings in post-treatment surgical specimens alone cannot establish pretreatment predictive value.

Potential explanations for discordant outcomes include epithelial suppression without sufficient matrix remodeling, or persistent prominence attributable to adipose tissue and skin redundancy. Neither explanation has been established by the studies reviewed here. These mechanistic hypotheses should be distinguished from endpoint selection, measurement methods, and assessment timing, which influence how response is detected and classified. Their clinical relevance should be investigated by linking measurements of the proposed intermediate changes with clinical outcomes in the same patients, rather than assigning a mechanism after the outcome is known. Changes from baseline alone do not establish comparative treatment benefit.

Bilateral gynecomastia offers a further opportunity to explore local factors associated with response. The two breasts share much of the systemic endocrine environment but may differ in local structure. Within-patient comparisons could help explore the contributions of systemic exposure and local tissue characteristics, provided that side-specific measurements are reliable and the two breasts are not analyzed as independent participants. Such comparisons could identify tissue features for larger prospective studies, while complementing rather than replacing between-patient treatment comparisons.

### 4.4. Aromatase Inhibition: Target Engagement and Clinical Efficacy

The randomized anastrozole trial illustrates the distinction between an endocrine effect and structural benefit. Eighty boys with pubertal gynecomastia received anastrozole or placebo for six months. Response was defined as a reduction of at least 50% in the combined volume of both breasts, calculated from ultrasound measurements. Response rates were 38.5% with anastrozole and 31.4% with placebo, with no statistically significant between-group difference despite a substantial change in the testosterone-to-estradiol ratio [18]. Anastrozole did not demonstrate superiority on the prespecified structural endpoint in the population studied. This result does not establish equivalence or exclude the possibility of benefit in particular subgroups.

The predefined ultrasound endpoint assesses breast volume and may be affected by measurement error; pain, satisfaction with appearance, and overall chest contour require separate assessment. The absence of a statistically significant between-group difference could reflect the sample size, population heterogeneity, disease duration, spontaneous regression, or endpoint sensitivity; the trial cannot distinguish these explanations. It therefore shows that superiority was not demonstrated on the prespecified structural endpoint, rather than proving equivalence or excluding benefit in a defined subgroup.

An open-label pharmacokinetic and pharmacodynamic study subsequently recruited 42 boys with recent-onset gynecomastia. It demonstrated aromatase blockade and reported reductions in breast area and volume from baseline [19]. These findings need not conflict with those of the randomized trial. Without a comparator, natural regression, maturation, and measurement variation may contribute to the observed improvement. Together, the studies distinguish three questions: whether drug exposure is achieved, whether the endocrine target is engaged, and whether treatment provides structural benefit beyond the expected untreated course. These questions should remain separate when interpreting endocrine responsiveness.

Earlier clomiphene studies also explored modification of the endocrine environment in adolescents [62,63]. These small clinical and endocrine investigations provide historical context, but do not identify a validated biological subgroup for treatment. Changes in systemic feedback may differ from the effects of modifying local receptor signaling. Comparisons should remain specific to the intervention, population, and outcome, rather than group all antiestrogen-related approaches into a single mechanistic class.

### 4.5. Tamoxifen: Clinical Evidence and Patient Selection

Two placebo-controlled crossover studies of tamoxifen included ten and six patients, respectively. They suggested benefit, particularly for tenderness, although evidence of structural change was less consistent in the smaller study [20,21]. Their small samples and short observation periods limit conclusions about variation in response. Pain relief and tissue shrinkage should be reported separately; combining them retrospectively into a single outcome may inflate the apparent response rate.

The short treatment windows and different crossover periods also limit direct comparison: the Parker study used one-month treatment periods, whereas the McDermott study used 2–4 months for each treatment period. These studies therefore provide signals for symptom and structural outcomes under short-term exposure rather than a validated duration or responder threshold.

Later comparisons with danazol or raloxifene, together with other clinical series, reported improvement in selected patients, although age, treatment indications, baseline severity, duration, and response definitions differed across studies [64,65,66,67]. In a prospective cohort of 81 men, Mannu and colleagues reported complete response in 73 following tamoxifen treatment [23]. This is an observational finding, not an estimate of benefit relative to observation alone. The ten-year period in the study title refers to the clinical recruitment period, not ten years of follow-up for each participant. In the absence of a concurrent comparator, spontaneous regression and the selection of symptomatic patients may also have contributed to the observed improvement.

Baseline lesion size illustrates how different endpoints can produce apparently conflicting findings. Devoto and colleagues associated larger lesions with a lower likelihood of complete disappearance, whereas a more recent retrospective adolescent series reported faster absolute reductions in disc diameter among patients with larger initial discs [68,69]. Earlier adolescent studies also characterized outcomes in relation to clinical size and treatment duration [70]. These findings are compatible: a larger lesion can shrink more in absolute terms yet remain less likely to disappear completely. Absolute change, proportional change, and complete resolution should be distinguished when considering size-based treatment thresholds.

Selection remains a concern in more recent observational comparisons. A study of 170 patients compared tamoxifen with surgery, but estrogen receptor assessment was confined to surgical patients rather than performed before treatment in both groups [24]. Its clinical outcomes and tissue findings cannot establish an ER-based rule for pretreatment selection. A separate retrospective study of 388 patients compared tamoxifen, Pingxiao capsules, and observation, with baseline clinical differences between groups [71]. The observation group provides information about the untreated course, but nonrandom treatment allocation complicates attribution of outcome differences to medication. These studies may identify candidate clinical predictors for validation; they do not establish modifiers of treatment effect.

### 4.6. Other Controlled Evidence and Treatment Safety

A randomized, double-blind trial of danazol enrolled 55 adults, of whom 52 were evaluated after protocol-related exclusions. Danazol improved tenderness and measures of enlargement compared with placebo, but significant weight gain occurred in the active-treatment group [22]. The findings show why symptom and structural outcomes need to be considered alongside harms. The distinction between the numbers enrolled and analyzed is also relevant when interpreting this older trial.

Small follow-up studies in adolescents and investigations of pubertal bone development have not identified a major safety signal with tamoxifen [72,73]. Their sample sizes, however, are insufficient to exclude uncommon or delayed adverse effects. A prospective cohort of men with breast cancer raised concerns about thromboembolism during tamoxifen treatment, but the older oncology population and treatment setting differ from short-course use in otherwise healthy adolescents [74]. Safety findings should be extrapolated cautiously in both directions. A biomarker that helps avoid unnecessary exposure could have clinical value even without identifying a subgroup with substantially greater treatment benefit.

### 4.7. Prevention Under a Defined Exposure

Trials of bicalutamide-associated breast events provide randomized evidence in a defined endocrine setting. In a trial of 114 men, gynecomastia occurred in 73% of those receiving bicalutamide plus placebo, 10% receiving bicalutamide plus tamoxifen, and 51% receiving bicalutamide plus anastrozole [25]. These studies address prevention of treatment-emergent breast events during active bicalutamide exposure, not reversal of established idiopathic gynecomastia. Other trials assessed tamoxifen dose, radiotherapy, and strategies for prevention or management [75,76,77]. These findings support receptor-directed intervention during a known, ongoing antiandrogen exposure. They do not establish that longstanding idiopathic gynecomastia is similarly reversible or justify extending prophylactic radiotherapy to otherwise healthy adolescents.

Prevention and reversal also place different demands on tissue remodeling. Preventing new enlargement does not require removal of an established stromal or adipose component. This may help explain why an intervention can be effective prophylactically yet produce less improvement in a pre-existing contour abnormality. It is a mechanistic interpretation of different clinical tasks, not evidence that fibrosis accounts for discrepancies between trials. Testing that explanation would require pretreatment tissue-related measurements and an explicit interaction analysis.

### 4.8. Implications of the Treatment Evidence

The treatment literature suggests that modifying endocrine action can improve selected breast outcomes, but the extent and meaning of improvement depend on the cause of gynecomastia, the intervention, and the endpoint assessed. The randomized aromatase-inhibitor trial shows why biochemical change alone cannot establish structural benefit. Small antiestrogen trials suggest symptomatic benefit, observational cohorts identify potentially response-associated clinical features, and prevention trials demonstrate efficacy under a defined exposure [18,20,21,23,25].

Across the therapeutic literature, age, developmental status, disease cause, symptom duration, and treatment intent should be treated as potential effect modifiers. Prevention during a defined antiandrogen exposure, treatment of recent-onset pubertal disease, and attempted reversal of established adult idiopathic gynecomastia are different clinical questions and should not be pooled as though they represented one treatment population.

An important next question is whether a pretreatment phenotype can distinguish patients likely to obtain meaningful additional benefit from medication from those whose principal residual concerns are structural or appearance-related. This is different from asking whether all patients should receive medication before surgery. Treatment effects need to be considered in relation to patient goals. Modest glandular regression accompanied by reliable pain relief may be valuable to one patient, while the same response may be inadequate for someone seeking correction of marked contour asymmetry. Patient preferences should be incorporated when evaluating a treatment strategy, rather than invoked retrospectively to explain its results.

Guideline recommendations also reflect the limitations of the evidence. The 2019 European Academy of Andrology guideline does not recommend selective estrogen receptor modulators or aromatase inhibitors for gynecomastia in general. The 2026 SIAMS guideline considers a short course of tamoxifen in selected recent-onset idiopathic cases, explicitly identifying the recommendation as expert opinion [1,2]. Neither guideline establishes a basis for receptor-guided prescribing. Further translational work should determine which measurable pretreatment characteristics help identify additional benefit, acceptable harms, and improvement in outcomes important to patients. Table 2 summarizes representative therapeutic studies, the outcome domains assessed, and their principal interpretive limitations.

## 5. Imaging and Patient-Derived Models as Translational Tools

Imaging and experimental models have complementary roles in the proposed framework. Imaging characterizes tissue and monitors change in patients, whereas experimental models allow functional hypotheses to be tested. Findings from either approach should be distinguished from patient-important benefit, and their use in treatment selection requires validation for the intended clinical purpose. A proposed, hypothesis-generating workflow integrating the two approaches is shown in Figure 2.

### 5.1. Diagnostic Imaging and the Unmet Need for Treatment Prediction

Treatment selection based on tissue characteristics generally requires measurements before surgery. Ultrasonography can describe the location and extent of glandular tissue without invasive sampling. Studies of sonographic appearances and the use of mammography and ultrasound in male breast disease provide a diagnostic foundation for developing longitudinal assessments [78,79,80]. Diagnosing gynecomastia, characterizing its tissue components, and predicting treatment benefit remain separate tasks. Accuracy in distinguishing benign from suspicious lesions does not establish an ability to predict the response to endocrine treatment.

The indication for imaging also needs to be considered. Studies of the additional value of ultrasound after mammographically confirmed gynecomastia, together with reports of possible imaging overuse, caution against assuming that more imaging necessarily improves care [81,82]. Studies of incidental gynecomastia on chest computed tomography (CT) describe patients who have already undergone imaging; they do not justify CT solely to characterize an otherwise benign presentation [83]. Research protocols should state what additional question each investigation addresses and distinguish research assessments from the clinically indicated diagnostic workup.

### 5.2. Tissue Stiffness and Histological Validation

Shear-wave elastography measures regional tissue stiffness rather than fibrosis itself. A prospective study assessed 92 breasts in 50 adolescents and found lower stiffness values in dendritic-pattern than in nodular-pattern gynecomastia [17]. This finding challenges the assumption that a dendritic pattern necessarily indicates a stiffer, more fibrotic lesion. The study did not establish a treatment-response threshold or validate its findings against paired histological classification of fibrosis. Its ability to distinguish imaging patterns does not, by itself, support the selection of patients for medication.

A next step would be to compare regional imaging findings with anatomically matched tissue from patients undergoing clinically indicated surgery. This could help determine whether stiffness reflects epithelial density, stromal organization, adipose admixture, or a combination of these features. Any resulting association would then require evaluation in a nonsurgical pretreatment cohort. These two stages would separate the development of a tissue surrogate from assessment of its treatment-predictive value, without assuming that surgical samples represent all patients presenting earlier in the disease course.

### 5.3. Reproducibility in Longitudinal Imaging

For longitudinal monitoring, an imaging measure must distinguish genuine change from variation due to patient positioning, probe pressure, segmentation, or the measurement plane. Maximum thickness, palpable diameter, and estimated volume are different endpoints and should not be used interchangeably. Agreement analysis, rather than correlation alone, is needed to assess repeatability and determine how much change can be distinguished from measurement error [84]. Two methods may correlate closely despite a clinically important systematic difference.

Protocols should prespecify patient positioning, anatomical landmarks, acquisition settings, the number of measurement repetitions, and criteria for defining the lesion. Bilateral measurements need to be analyzed with their within-patient correlation taken into account. Where feasible, blinded reassessment can help distinguish variation in image interpretation from biological change. These are proposed safeguards, not a validated imaging standard for gynecomastia. Reliable acquisition and interpretation remain necessary even when the measurements are incorporated into a complex prediction model.

Imaging studies should also specify which tissue component changes. The retroareolar glandular region may shrink without a corresponding reduction in overall breast prominence, while changes in body weight may alter the adipose component independently of medication. Component-specific imaging, assessed alongside symptoms and appearance-related scores, could clarify these discrepancies rather than leave them classified as unexplained treatment failure. The aim is to obtain reproducible measurements that inform clinical interpretation or management, rather than to maximize imaging detail.

### 5.4. Gynecomastia-Derived Organoids

A 2025 study described a protocol for generating gynecomastia-derived organoids and reported cultures from six patients with physiological, idiopathic, or hormone-related presentations. The cultures formed gland-like structures and retained selected epithelial and proliferative markers. Immunohistochemistry was assessed across six donor-derived cultures, whereas immunofluorescence and quantitative PCR (qPCR) were performed in two selected cases (GYN-1 and GYN-4). In these two cases, organoids showed reduced ERα immunofluorescence signal, while qPCR detected no significant difference in ESR1 transcript levels relative to matched tissues. Endocrine responsiveness was not tested using estrogen or androgen stimulation [10]. The cultures came from only six biological donors, the study supports feasibility and model characterization rather than population-level reproducibility or prediction of clinical drug response.

Earlier studies of estrogen receptor transcripts in gynecomastia tissue and primary cultures, together with work on regenerative capacity in nonmalignant mammary culture systems, support the distinction between establishing a culture and preserving the biology of the source tissue [52,85,86]. Culture conditions favor cells capable of surviving and expanding under those conditions. The resulting population may be suitable for experiments yet differ from the tissue compartment responsible for symptoms or limited regression. Culture success and biological representativeness need to be assessed separately.

Reference atlases and comparative studies can assist model characterization, although they cannot replace validation in gynecomastia. A spatially resolved atlas of the adult human breast provides a reference for identifying cellular compartments and anatomical relationships [87]. A 2026 single-cell study comparing testosterone-exposed breast tissue from transgender men with tissue from cisgender women reported reduced estrogen-associated signaling in hormone receptor-positive luminal cells despite similar circulating estradiol concentrations [88]. This illustrates possible discordance between systemic hormone concentrations and local transcriptional activity. It is not, however, direct evidence about gynecomastia in cisgender men or a basis for androgen treatment in eugonadal patients.

### 5.5. Functional Assessment of Endocrine Responsiveness

An endocrine-response assay would compare baseline and stimulated conditions, document a receptor-linked response, and test whether a relevant perturbation reverses that response. Assessment should extend beyond aggregate metabolic viability. Candidate readouts include lineage-resolved proliferation, ductal organization, hormone-responsive transcription, and matrix remodeling where the model permits it. The choice and interpretation of endpoints depend on the tissue components represented. An epithelial-only organoid may allow receptor dependence to be tested but cannot, by itself, explain how stromal constraints limit improvement in chest contour.

Drug metabolism also needs to be considered. Pharmacological studies of tamoxifen have identified active metabolites and the importance of sequential cytochrome P450-mediated biotransformation [89,90]. A culture without the relevant metabolic capacity may respond to the parent drug differently from a patient’s tissue exposed to circulating metabolites. Experiments should specify the compound or metabolite tested, justify the exposure range, and distinguish receptor-linked activity from nonspecific toxicity. These considerations improve experimental interpretation; they do not establish a basis for routine pharmacogenetic testing or organoid-guided tamoxifen prescribing in gynecomastia.

No direct evidence currently shows that gynecomastia-derived organoids reproduce the relevant CYP450 metabolic capacity. The metabolism discussion should therefore be understood as a methodological consideration for future model validation, rather than as an established feature of the described cultures.

Co-culture may be useful for studying epithelial–stromal interactions or local steroid conversion. Fibroblastic, adipose-associated, or other relevant components could be introduced under controlled conditions and compared with the epithelial model alone. Each added component would need to make a defined contribution to the experiment and be assessed for the variability it introduces. For questions involving the matrix, tissue slices or defined-matrix systems could complement organoids by retaining or controlling relevant structural features. These remain proposed approaches rather than established gynecomastia-specific assays.

The ERα immunofluorescence and ESR1 transcript findings in these two cases warrant further investigation during model validation. Transcript abundance alone does not establish receptor signaling, and reduced immunofluorescence signal does not reveal its cause. Differences in cell composition, translation, protein stability, or sampling could account for this pattern. Cell-level comparisons between matched tissue and early-passage cultures could help distinguish these explanations before drug testing. Subsequent stimulus–response time-course experiments could assess whether the relevant downstream responses are retained, attenuated, or lost [10,52,86].

### 5.6. Sampling and Patient-Level Reproducibility

The source of a model is as important as the culture method. Surgical specimens are enriched for persistent, conspicuous, distressing, or previously treated disease and may underrepresent early lesions that regress or respond to medication. A model bank should record symptom duration, developmental stage, prior treatment, sampling region, and relevant endocrine exposures, rather than classify every sample simply as gynecomastia. Tissue-processing details should also be documented to distinguish biological variation from differences in specimen handling.

Replication must be defined at the appropriate level. Wells, organoids, microscopic fields, and repeated assays do not represent independent patients. Multiple wells can improve the estimate of within-patient experimental variation, but do not increase the number of independent patient samples. Candidate responses need to be reproduced in cultures derived from different patients, and associations with clinical outcomes should be evaluated at the patient level. Models derived only from patients undergoing surgery after unsuccessful treatment may help characterize resistance-associated features, but cannot establish their ability to predict treatment sensitivity without an appropriate comparison population.

Patient-derived models may be most useful in the near term for investigating mechanisms rather than assigning individual treatments. They can test the functional relevance of tissue-associated signals and help identify accessible correlates for subsequent clinical evaluation. This would connect findings from surgical material with pretreatment assessment without requiring tissue sampling from every future patient. Negative findings are also informative: failure to retain receptor responsiveness should be recognized as a potential model limitation before it is interpreted as biological resistance in the patient. Table 1 summarizes representative tissue, imaging, and patient-derived model studies, together with their translational contributions and limitations.

## 6. Discussion and Future Directions

The reviewed evidence suggests that endocrine responsiveness and the capacity of established tissue to regress should be considered separately. Receptor studies identify plausible signaling components, while histological investigations describe variation in epithelial and stromal composition [9,14,16,59]. The anastrozole trial illustrates the limits of inferring structural benefit from biochemical effects: treatment altered the hormonal environment but did not demonstrate superiority over placebo on the prespecified structural endpoint [18]. Whether tissue composition or remodeling capacity contributes to this discordance remains a hypothesis requiring direct testing.

Further research should link pretreatment measurements to two questions in gynecomastia: whether medication offers additional symptom or structural benefit over observation, and how surgical treatment of persistent contour concerns affects outcomes important to patients. Both questions require a defined developmental and etiological context. Patient-derived models and paired tissue–imaging studies may support this research, although the available gynecomastia organoid study has not established endocrine responsiveness or concordance with patient outcomes [10].

### 6.1. Defining and Measuring Patient-Important Benefit

The intended benefit should be specified before selecting a marker or experimental readout. Psychological and quality-of-life studies show that lesion dimensions alone do not capture the burden of gynecomastia [6,7,8]. Pain relief, reduced appearance-related distress, and improved daily functioning may each be relevant, but require measures suited to the outcome and population. The BODY-Q chest module was developed for chest-contouring outcomes in a population that included patients with gynecomastia [91]. Its use in other languages, age groups, or pharmacological settings requires attention to content relevance, responsiveness, and interpretability. COSMIN guidance provides a framework for assessing these measurement properties [92].

A pragmatic design for future comparative trials would place a patient-important outcome at the primary endpoint level and treat biological and structural measurements as prespecified secondary or mechanistic endpoints. For pharmacological treatment of symptomatic recent-onset disease, pain or tenderness and appearance-related distress could be assessed alongside component-specific imaging; for surgery, contour and satisfaction with appearance may be more central. These are proposed design principles rather than validated outcome standards. Assessments should include baseline, end-of-treatment, post-discontinuation, and longer-term follow-up when persistence of benefit is clinically relevant.

Outcome assessment should distinguish early symptom relief, structural change during treatment, persistence of benefit after discontinuation, and longer-term appearance-related well-being. Assessment times should reflect the clinical context rather than follow a universal schedule. Subsequent surgery also needs to be reported with its indication: residual contour concerns, persistent pain, recurrent glandular enlargement, and patient preference are not interchangeable reasons for intervention. Linking these observations with tissue and model findings would help determine whether a response in one domain corresponds to improvement in another.

### 6.2. Linking Tissue Findings to Pretreatment Assessment

An initial discovery study could pair clinical phenotype and imaging with multiregion tissue measurements in patients already undergoing clinically indicated surgery. Prespecified sampling and blinded evaluation would help determine whether an imaging feature reflects the tissue process of interest. Principles for evaluating archived specimens and reporting biomarker studies are relevant, with adaptation to the sampling and clinical context of a benign condition [12,93]. The aim at this stage is to identify reproducible associations and candidates for functional testing, rather than establish a prescribing threshold.

Markers suitable for pretreatment measurement could then be evaluated in consecutively recruited patients before treatment selection. Their added value should be assessed against a baseline clinical evaluation that includes developmental stage, symptom duration, pain, lesion dimensions, and etiological context. Statistical significance alone would not demonstrate added clinical value.

### 6.3. Distinguishing Treatment Response from Treatment Benefit

A model developed in a single treated cohort estimates the probability of an outcome in that treatment setting. It does not show how much improvement is attributable to treatment rather than spontaneous regression or other changes over time. Patients predicted to improve with medication may also have a favorable untreated course. Prediction of treatment benefit requires comparison between treatment strategies and assessment of whether the benefit differs according to the marker [11].

Randomized comparisons with prespecified analyses of effect modification provide a stronger basis for this assessment. The credibility of subgroup findings depends on their prespecification, the information available to support the comparison, and whether thresholds were selected after examining the results. ICEMAN provides a framework for evaluating these issues [94]. Treatment effects should be reported with uncertainty on clinically interpretable scales. A significant result in one subgroup and a nonsignificant result in another do not, by themselves, establish a difference in treatment effect.

### 6.4. Model Development and Validation

The number of candidate predictors should be proportionate to the information available from independent patients. Sample-size planning needs to account for the outcome distribution, number of model parameters, and anticipated performance [95]. TRIPOD+AI reporting guidance and PROBAST+AI assessment principles are relevant to both regression-based and machine learning models [96,97]. The choice of algorithm does not resolve problems arising from selected recruitment, unreliable measurements, or poorly defined outcomes.

Validation should assess calibration as well as discrimination. Measurements from both breasts and repeated visits for the same patient must remain together when separating development and evaluation datasets. External validation should examine performance across age groups, centers, assay platforms, and treatment practices. Missing data and the selective availability of pathology require explicit consideration; tissue findings from surgical patients cannot be assumed to represent patients who never undergo tissue sampling. These issues are particularly important when a model developed from persistent or previously treated disease is intended for use earlier in the clinical course.

### 6.5. Evaluating Clinical Utility

A prediction may be accurate without improving care. Decision-curve analysis can assess net benefit across relevant thresholds, but does not replace a clinical impact study [98]. A marker-guided strategy should ultimately be compared with usual clinical assessment, taking account of unnecessary treatment, missed opportunities for benefit, testing burden, and patient preferences. In gynecomastia, clinical value may include avoiding ineffective medication or additional invasive investigations, rather than increasing the proportion of patients treated.

In gynecomastia, the proposed sequence is a long-term research agenda rather than an immediately implementable clinical pathway. Each stage may require multicenter recruitment, stable imaging and tissue assays, and patient-level validation before it can support a treatment decision.

Both the marker-based decision rule and the management that follows from it should be specified before enrollment. Otherwise, differences in clinician discretion or follow-up intensity may obscure the contribution of the marker-guided strategy. Trial reports should clearly describe allocation, outcomes, harms, and missing observations, following applicable guidance such as CONSORT 2025 [99]. Transparent reporting supports interpretation but does not, by itself, establish methodological quality.

Table 3 and Figure 3 summarize the proposed stages of research, from tissue discovery and functional investigation to pretreatment assessment and clinical evaluation. These are stages in the development of a clinical test, not a required sequence of biological, structural, and patient-reported changes within an individual patient.

## 7. Limitations

This critical narrative review synthesizes biological, clinical, and methodological evidence but does not provide a systematic estimate of treatment effects. Focused searches and reference tracing may have missed relevant studies, including non-English publications and work not covered by the indexing sources searched. Independent duplicate screening, formal risk-of-bias grading, and quantitative synthesis were not undertaken. For some publications, assessment was limited to indexed abstracts, restricting appraisal of experimental methods and potential sources of bias.

The evidence supporting individual components of the framework varies in strength. The gynecomastia-derived organoid study established a disease-specific model but did not establish endocrine responsiveness or a relationship between model responses and patient outcomes [10]. The imaging studies reviewed here have not validated a link between pretreatment imaging features, histological tissue state, and subsequent treatment benefit [17,78,79,80,81,82,83]. Mechanistic and methodological work in other breast systems can inform study design, but does not replace validation in gynecomastia.

The three-domain framework is an interpretive proposal rather than an empirically derived classification. Its usefulness will depend on whether it helps clarify discordant outcomes and generate testable research questions. This review focuses on translation and treatment selection, rather than comprehensive clinical guidance. It does not provide a treatment algorithm, a comparative review of surgical approaches, or recommendations for additional routine investigations. The research priorities outlined here should not be interpreted as established standards of care.

## 8. Conclusions

In gynecomastia, biological activity, structural change, and patient-important benefit should be assessed separately and interpreted together. Human genetic studies and evidence from defined endocrine exposures support endocrine causation in selected settings, while tissue studies describe frequent receptor expression alongside structural heterogeneity. Treatment studies show that biochemical changes and improvement within a treated group do not, by themselves, establish additional benefit over observation or alternative management.

Ultrasonography may support diagnostic assessment and characterization of tissue extent when clinically indicated. The use of receptor assays, imaging features, or organoid drug-sensitivity assays as predictors of differential treatment benefit remains investigational and requires validation before marker-guided treatment selection.

Further progress depends on connecting these findings to clinical assessment, rather than replacing it with an unvalidated molecular classification. Paired tissue and imaging studies can identify candidate pretreatment measures, patient-derived models can test functional mechanisms, and comparative clinical studies can assess whether those measures identify differences in treatment benefit. Each of these links requires validation; none implies that patients must follow a fixed sequence of biological, structural, and patient-reported changes. Clinical utility ultimately depends on reproducible improvements in patient-important outcomes or treatment decisions, weighed against harms, testing burden, and resource use. The aim is to connect what is measured with the process being treated and the outcome the patient seeks.

## Figures and Tables

**Table 1:** Representative tissue, imaging, and patient-derived model evidence in gynecomastia.

Study and Material	Principal Finding	Translational Relevance	Key Interpretive Limitation	Evidence Category
Prasad et al. (2022) [9] Tissue profiling; 86 patients, 108 evaluable specimens.	ERα positivity in approximately 99% of evaluable specimens; receptor and cytokeratin patterns were characterized.	Identifies a receptor-rich epithelial compartment that warrants functional testing.	Cross-sectional surgical material; receptor positivity alone does not establish functional dependence or treatment-predictive value.	Primary human tissue study
Singh et al. (2022) [14] Surgical tissue study; 29 men with bilateral moderate-to-severe idiopathic disease.	Frequent steroid-receptor and aromatase expression; IGF-related signals were investigated.	Documents steroid receptor and IGF-1 expression in selected surgical specimens.	Selected surgical population; expression does not establish functional dependence or differential treatment benefit.	Primary human tissue study
Fricke et al. (2018) [15] Cross-sectional histology; 305 patients, 555 breasts.	Fibrous appearances were more common in younger surgical groups.	Shows fibrous patterns occur across age groups, not only in older patients.	Cross-sectional design; surgical selection bias.	Primary human histology study
Lanzafame et al. (1994) [16] Collagen distribution study; 36 specimens.	Type VI collagen was prominent in florid lesions but reduced or absent in densely fibrotic regions.	Type VI collagen distribution differs between florid and fibrotic regions.	No direct test of drug response or structural reversibility.	Primary human tissue study
Akgül et al. (2012) [13] Post-treatment case report; 3 adolescents undergoing surgery after tamoxifen.	Despite persistent dissatisfaction, resected tissue contained adipose tissue without intraductal epithelial proliferation.	Raises possible discordance between tissue findings and patient satisfaction.	Post-treatment cases only; no pretreatment comparison.	Primary human post-treatment case report
Parlak et al. (2025) [17] Prospective elastography study; 50 adolescents, 92 breasts.	Dendritic-pattern lesions had lower stiffness than nodular-pattern lesions.	Lower stiffness in dendritic-pattern versus nodular-pattern gynecomastia.	No validated link to histological fibrosis or treatment benefit; stiffness is not an established treatment-selection threshold.	Primary human prospective imaging study
Shang et al. (2025) [10] Patient-derived organoid study; cultures from 6 patients.	Selected structures and epithelial markers were retained; reduced ERα protein coexisted with relatively preserved *ESR1* transcripts.	Gynecomastia-derived organoids retain gland-like structures and epithelial markers.	Endocrine stimulation responses and concordance with patient outcomes were not established.	Primary human patient-derived model study

Representative primary studies selected from disease-specific literature; this was not a systematic review with exhaustive searching or formal risk-of-bias grading. Patient counts are distinguished from breast, specimen, and culture counts.

**Table 2:** Representative therapeutic evidence and interpretive limits.

Study, Design, and Evidence Category	Population and Comparison	Outcomes and Principal Findings	Translational Interpretation
Primary human randomized comparative study—Plourde et al. (2004) [18] Randomized, double-blind, placebo-controlled trial.	80 boys with pubertal gynecomastia. Anastrozole vs. placebo; 6 months.	Structural response ^a^: 38.5% vs. 31.4%; no significant between-group difference despite a substantial change in the testosterone-to-estradiol ratio.	Biochemical target engagement did not establish structural superiority. A nonsignificant result does not demonstrate equivalence.
Primary human prospective open-label study—Mauras et al. (2009) [19] Open-label PK/PD study.	42 boys recruited with recent-onset disease. Anastrozole 1 mg daily for 6 months; PK/PD assessed after 14 days; no concurrent comparator.	Exposure and biological activity: aromatase blockade and within-group reductions in breast area and volume were reported.	Demonstrates biological activity, not added benefit beyond natural regression, maturation, or measurement variation.
Primary human randomized crossover studies—Parker et al. (1986) [20] McDermott et al. (1990) [21] Placebo-controlled crossover trials.	10 and 6 patients, respectively. Parker: tamoxifen and placebo periods of 1 month; McDermott: 2–4 months for each period. Tamoxifen vs. placebo. Parker: 8/10 patients were reassessed 9–12 months after treatment cessation.	Symptoms and structure: benefit signals, particularly for tenderness; less consistent structural findings in the smaller study.	Small samples and short observation limit estimation of structural benefit and reliable responder subgroups.
Primary human randomized comparative study—Jones et al. (1990) [22] Randomized, double-blind, placebo-controlled trial.	55 adults enrolled; 52 evaluated. Danazol 200 mg twice daily for 3 months vs. placebo.	Symptoms, structure, and harms: tenderness and enlargement measures improved; significant weight gain occurred with danazol.	Benefits and harms require joint assessment. Enrollment and analysis denominators must remain distinct.
Primary human prospective uncontrolled cohort—Mannu et al. (2018) [23] Prospective treatment cohort.	81 men. Tamoxifen 10 mg daily; mean treatment duration 7 months (range 1–25 months); no concurrent comparator.	Study-reported complete response ^b^: observed in 73 of 81 patients.	An uncontrolled response proportion is not a comparative treatment-effect estimate; spontaneous regression and selection remain alternative explanations.
Primary human retrospective comparative cohort—He et al. (2023) [24] Retrospective treatment comparison.	170 patients. Tamoxifen 10 mg daily for 3–6 months vs. surgery; follow-up within 6 months after tamoxifen discontinuation or surgery.	Clinical outcomes and receptor assessment: outcomes were compared; ER testing was confined to surgical patients.	Nonrandom treatment choice and surgical-group-only ER assessment do not validate pretreatment ER-guided treatment selection.
Primary human randomized prevention trial—Boccardo et al. (2005) [25] Randomized prevention trial.	114 men receiving bicalutamide for 48 weeks. Placebo, tamoxifen, or anastrozole.	Prevention: gynecomastia occurred in 73%, 10%, and 51% with placebo, tamoxifen, and anastrozole, respectively.	Exposure-specific prevention does not establish reversal of longstanding idiopathic gynecomastia.

Results are presented in their original clinical settings and not pooled. (^a^) Response was defined in the original trial as a ≥50% reduction in combined ultrasound-estimated breast volume at 6 months. (^b^) Complete response was defined as complete symptom resolution assessed by the patient and surgeon; this uncontrolled proportion should not be interpreted as a comparative treatment effect. PK/PD, pharmacokinetic/pharmacodynamic; ER, estrogen receptor.

**Table 3:** Proposed translational research sequence and evidence requirements for progression.

Research Stage	Primary Question	Suggested Study Approach	Evidence Required for Progression
1. Clinical phenotyping	What process and patient burden require treatment?	Record developmental stage, exposure history, symptoms, and component-specific dimensions; define biological, structural, and patient-important outcomes separately.	Explicit population and endpoint definitions; record age, symptom duration, and tissue state separately rather than assigning an unvalidated molecular label.
2. Tissue–imaging pairing	Does an accessible imaging feature reflect the proposed tissue process?	Pair regional imaging with anatomically matched, multiregion specimens from patients already undergoing indicated surgery; use prespecified sampling and blinded assessment.	Reproducible measurement, anatomical concordance, and transparent handling of surgical selection before evaluation in nonsurgical pretreatment cohorts.
3. Functional validation	Does the candidate process respond to a defined, mechanistically relevant perturbation?	Compare matched tissue and models; perform stimulus-response experiments, receptor-directed perturbation, and independent molecular readouts.	Replication across independent patients, retention of relevant cellular compartments, and separation of receptor-linked effects from nonspecific toxicity.
4. Pretreatment prediction	Does the marker add information beyond routine clinical assessment?	Evaluate consecutively recruited pretreatment cohorts against a prespecified clinical baseline; assess calibration, discrimination, and external performance.	Stable assays, adequate sample size, patient-level separation of development and validation data, and explicit handling of missing data and selective tissue availability.
5. Treatment-benefit evaluation	Does the marker identify differential benefit between reasonable treatment options?	Use comparative, preferably randomized studies with prespecified marker-by-treatment interaction analyses and clinically interpretable treatment-effect estimates.	Credible effect modification with uncertainty reported; neither improvement in one treated cohort nor significance in only one subgroup establishes differential benefit.
6. Clinical impact	Does using the marker improve patient-important decisions and outcomes?	Compare a prespecified marker-guided strategy with usual assessment; include patient-important outcomes, harms, testing burden, and resource use.	Demonstrated net benefit and feasible implementation

This is a proposed, long-term research agenda for developing clinical tests, not a validated classification or treatment algorithm. The stages do not imply that biological, structural, and patient-reported responses must follow a fixed sequence in individual patients. Methodological references are discussed in the text [11,12,93,94,95,96,97,98,99].

## Data Availability

No new data were created or analyzed in this study. Data sharing is not applicable to this article.

## Abbreviations

The following abbreviations are used in this manuscript:

CAG	cytosine–adenine–guanine
CD34	cluster of differentiation 34
CONSORT	Consolidated Standards of Reporting Trials
COSMIN	Consensus-based Standards for the Selection of Health Measurement Instruments
CT	computed tomography
CYP19A1	cytochrome P450 family 19 subfamily A member 1
ER	estrogen receptor
ESR1	estrogen receptor 1
GPR30	G protein-coupled receptor 30
IGF-1	insulin-like growth factor 1
PROBAST	Prediction model Risk Of Bias ASsessment Tool
SIAMS	Italian Society of Andrology and Sexual Medicine
TGF-β	transforming growth factor beta
TRIPOD	Transparent Reporting of a multivariable prediction model for Individual Prognosis Or Diagnosis
AIs	aromatase inhibitors
AR	androgen receptor
BODY-Q	Breast-Q-derived chest outcome module
ECM	extracellular matrix
ERα	estrogen receptor alpha
ERβ	estrogen receptor beta
IHC	immunohistochemistry
PK/PD	pharmacokinetic/pharmacodynamic
SERMs	selective estrogen receptor modulators
ICEMAN	Instrument for assessing the Credibility of Effect Modification Analyses
